# Would Parents Get Their Children Vaccinated Against SARS-CoV-2? Rate and Predictors of Vaccine Hesitancy According to a Survey over 5000 Families from Bologna, Italy

**DOI:** 10.3390/vaccines9040366

**Published:** 2021-04-10

**Authors:** Marco Montalti, Flavia Rallo, Federica Guaraldi, Lapo Bartoli, Giulia Po, Michela Stillo, Paola Perrone, Lorena Squillace, Laura Dallolio, Paolo Pandolfi, Davide Resi, Maria Pia Fantini, Chiara Reno, Davide Gori

**Affiliations:** 1 Unit of Hygiene, Department of Biomedical and Neuromotor Sciences, Public Health and Medical Statistics, University of Bologna, 40126 Bologna, Italy; flavia.rallo@studio.unibo.it (F.R.); laura.dallolio@unibo.it (L.D.); mariapia.fantini@unibo.it (M.P.F.); chiara.reno@studio.unibo.it (C.R.); davide.gori4@unibo.it (D.G.); 2 IRCCS Istituto delle Scienze Neurologiche di Bologna, 40139 Bologna, Italy; federica.guaraldi@yahoo.it; 3 Unit of Primary Health Care, Department of Medical and Surgical Sciences, University of Bologna, 40126 Bologna, Italy; lapo.bartoli@studio.unibo.it; 4 School of Hygiene and Preventive Medicine, University of Ferrara, 44121 Ferrara, Italy; giulia.po@edu.unife.it; 5 Department of Public Health, Bologna Local Health Authority, 40124 Bologna, Italy; michela.stillo@ausl.bologna.it (M.S.); paola.perrone@ausl.bologna.it (P.P.); lorena.squillace@ausl.bologna.it (L.S.); paolo.pandolfi@ausl.bologna.it (P.P.); davide.resi@ausl.bologna.it (D.R.)

**Keywords:** vaccine hesitancy, children, vaccination, COVID-19, SARS-CoV-2, survey

## Abstract

In the near future, COVID-19 vaccine efficacy trials in larger cohorts may offer the possibility to implement child and adolescent vaccination. The opening of the vaccination for these strata may play a key role in order to limit virus circulation, infection spreading towards the most vulnerable subjects, and plan safe school reopening. Vaccine hesitancy (VH) could limit the ability to reach the coverage threshold required to ensure herd immunity. The aim of this study was to investigate the prevalence and determinants of VH among parents/guardians toward a potentially available COVID-19 vaccination for children and adolescents. An online survey was performed in parents/guardians of children aged <18 years old, living in Bologna. Overall, 5054 questionnaires were collected. A vast majority (60.4%) of the parents/guardians were inclined to vaccinate, while 29.6% were still considering the opportunity, and 9.9% were hesitant. Highest vaccine hesitancy rates were detected in female parents/guardians of children aged 6–10 years, ≤29 years old, with low educational level, relying on information found in the web/social media, and disliking mandatory vaccination policies. Although preliminary, these data could help in designing target strategies to implement adherence to a vaccination campaign, with special regard to web-based information.

## 1. Introduction

Since March 2020, most governments around the world have closed schools and other educational institutions in an attempt to contain the diffusion of the COVID-19 pandemic, with an estimated dramatic impact on the education of over 850 million children and youths [1,2,3,4]. On the other hand, epidemiological data show that people aged 1–18 years tend to develop asymptomatic/paucisymptomatic disease, and, overall, present a significantly more favorable outcome than adults [5,6,7]. Furthermore, younger children seem to be less susceptible to infection and transmitting the disease compared to older children, adolescents, and adults [3,5]. However, with the emergence of new variants, the risk of disease transmission and outcomes in children deserve close surveillance [5,6,7].

In Italy, school activities were suspended on 4 March 2020, based on the epidemiological evolution and the progressive viral spread on the national territory. The initial suspension turned into a protracted closure due to the subsequent lockdown of the whole country [8], and the educational services resumed their activities only in mid-September of 2020. As of 5 January 2021, 262,654 positive cases were recorded in the school population (0–19 years old) since the beginning of the pandemic [9], posing challenges for both the educational and the National Health System (SSN) [10].

In the Emilia-Romagna Region (ERR), positive cases of SARS-CoV-2 within the school community have been tracked since the resumption of educational service activities through a dedicated screening system operated by the Local Health Authority, with the aim of identifying and managing any possible outbreak within classes and schools. Specifically, the entire class is put into quarantine every time in case of outbreak (>1 case), or when a single positive case occurs in a community with a high risk of COVID-19 transmission (e.g., children aged <6 years, for whom the use of personal protective equipment (PPE) is not mandatory] [11]. These measures cause extreme discontinuity in educational services with important logistic consequences for the children and their families, as well as costs and for workload for the local health care system to test the index case and all his/her contacts.

In December 2020, the European Centre for Disease Prevention and Control (ECDC), based on the data on the experience of school closure in European countries from August to December 2020, recommended to consider this measure as a last resort in the pandemic management [12]. Accordingly, the Italian Government decided to suspend teaching activities only in the areas at higher risk of COVID-19 transmission, that is, with higher incidence of cases per inhabitant, and number of beds occupied in intensive care units. Accordingly, teaching services switched to remote learning on 1 March 2021 in Emilia-Romagna [13].

At the end of December 2020, the vaccination campaign against SARS-CoV-2 began in Italy, initially involving healthcare professionals and residents of Long-Term Care Facilities (LTCFs) considered at high risk of exposure and severe outcomes, respectively. The vaccination schedule intended to involve teachers and school staff as early as the second phase of the vaccination campaign [14]. Children and adolescent vaccination could play a primary role to limit the circulation of the virus, the spread of the infection to the most vulnerable subjects, and to plan safe school reopening. Therefore, the efficacy and safety of COVID-19 vaccines in people aged <18 years old are currently being tested [15]. On the other hand, Vaccine Hesitancy (VH), defined as a “delay in acceptance or refusal of vaccination despite availability of vaccination services” [16], could limit the diffusion and, therefore, the ability to reach the coverage threshold required to ensure the efficacy of the COVID-19 vaccine.

The aim of this study was to investigate the prevalence and determinants of VH among parents/guardians toward COVID-19 vaccination in children and adolescents, so as to orient future awareness-raising tools and vaccination strategies.

## 2. Materials and Methods

An online survey was performed in parents/guardians of children aged <18 years old, living in the Metropolitan City of Bologna, recruited between December 2020 and January 2021 among family members interviewed by the personnel of the local Public Health Service dedicated to school-based SARS-CoV-2 screening. Recruitment was obtained through emails sent to principals, who were in charge of dissemination. Adhesion to the study was voluntary; data were collected anonymously. All participants provided online informed consent to be included in the database for study participation.

### 2.1. Questionnaire

Socio-demographic variables of respondents and their children were collected. Participants were also asked whether their children had received mandatory vaccinations (i.e., polio, diphtheria, tetanus, hepatitis B, pertussis, Haemophilus influenzae type B, measles, rubella, mumps and varicella, L. 119/2017 [17]) and if they would have their children vaccinated against SARS-CoV-2. VH determinants (i.e., medical advice, personal belief, web/social media, and television as sources of information to make a decision on the COVID-19 vaccination), chosen according to those identified by the WHO Sage Group [18] were investigated. Finally, participants were asked to indicate their level of agreement with the statement: “The Italian government (“Lorenzin” Law, n. 119/2017), in order to prevent communicable diseases and reduce public health concerns, increased from 4 to 10 the number of obligatory vaccines in children to be enrolled in nurseries and kindergartens”, and “Your views on vaccination requirements have changed because of the current SARS-CoV-2 (COVID-19) pandemic”, using a 5-level Likert rating scale. The English version of the survey can be found in the Supplementary File S1.

### 2.2. Statistical Analysis

Variables were described as absolute frequencies and percentages. Determinants of VH were assessed by uni- and multivariate analyses. Results of multivariate analyses are presented as an odds ratio (OR) with standard error (SE) and a 95% confidence interval (95% CI). A backward stepwise analysis was run to define the variables to be included in the final multiple logistic regression model, according to the results of univariate models and to the principles of parsimony and biological plausibility. Statistical significance was set at *p* < 0.05. Data were collected using Microsoft Excel (Microsoft Corporation). All analyses were carried out using Stata Statistical Software 15 (StataCorp, College Station, TX, USA).

## 3. Results

### 3.1. Sample Features

Overall, 5054 questionnaires were collected. Fifty-four had to be excluded as participants denied the consent to the treatment of personal data, and seven for non-compliant data (children’s age and school grade were not consistent), leaving 4993 questionnaires available for data collection and analysis. The main parent/guardian demographic and education data are reported in Table 1a; child distribution by age is reported in Table 1b.

Of the total respondents, 76.6% (*N* = 3824) were females; the great majority (*N* = 2764, 55.4%) were aged between 40 and 49 years old, while few (*N* = 89, 1.8%) were younger than 30. With regard to the level of education, most (*N* = 2275, 45.6%) had a high-school qualification, followed by those with a university degree (*N* = 1654, 33.1%), a post-university Master’s degree (*N* = 571, 11.4%), and finally, those with a primary school qualification (*N* = 493, 9.9%) (Table 1a). Analysis of age distribution among children demonstrated the prevalence of children aged 6–10 years (1807, 36.2%), followed by those aged 11–13 (*N* = 1312, 26.3%), ≥14 (*N* = 1241, 24.9%) and, finally, 0–5 years (*N* = 633; 12.7%) (Table 1b).

### 3.2. Attitude toward COVID-19 Vaccination and Main Determinants

The exploration of adherence to Italian obligatory vaccination law resulted in 29 (0.6%) non-adherent, 118 (2.4%) partially adherent, and 4846 (91.1%) fully adherent.

When exploring the inclination to have their children receive a COVID-19 vaccination, the results show that 496 (9.9%) did not intend to vaccinate their children, 1480 (29.6%) did not know, and 3017 (60.4%) were inclined to have their children receive a COVID-19 vaccination.

Sources of information chosen by parents/guardians to decide regarding COVID-19 vaccination in children are reported in Table 2. Most of the patients relying on medical advice were prone to vaccinating, while those following personal beliefs, web/social media, or celebrities were hesitant.

According to multivariate regression, age, gender, and level of education of parents/guardians, children’s age, information source(s), as well as attitudes toward mandatory vaccination policy before and after the COVID-19 pandemic significantly correlated to VH (*p* < 0.05) (Table 3). Specifically, the highest OR of VH were detected in female parents/guardians of children aged 6–10 years old, ≤29 years old, with low educational level, relying on information found in the web/social media, and disliking mandatory vaccination policies. On the other hand, attitudes toward mandatory vaccination changed after the COVID-19 pandemic. A total of 474 respondents liking the mandatory vaccination policies stated that they changed their opinion due to the pandemic, whereas conversely, 65 parents/guardians began to agree with mandatory policies (data not shown).

Of the four SAGE determinants, we studied those which were the most and least protective for VH in relation to the level of parents/guardians’ education (Table 4). It can be observed that those who considered medical advice less important (*n* = 358, 72.6%) had a lower level of education, whereas those who had completed postgraduate studies had a 90% level of reliance on physicians (*n* = 514). Moreover, 6% (*n* = 34) of the most educated people used the Web and/or Social Media as a principal source of information to decide about vaccination (Table 4).

### 3.3. Adherence and Perception of the Policy on Obligatory Vaccination (Law n. 119/2017)

**Figure 1:**
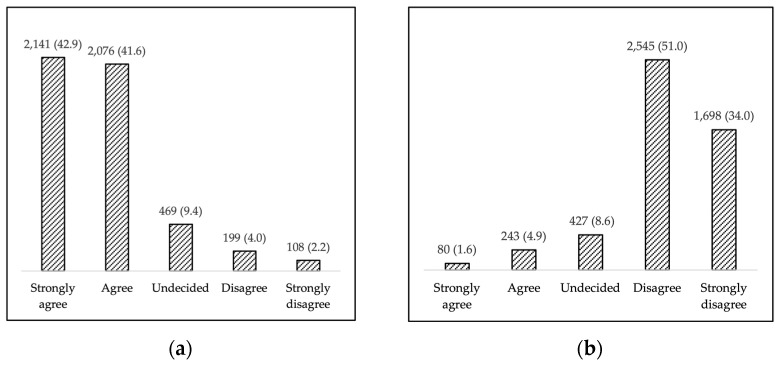
(**a**) Level of agreement with mandatory vaccination policies (Decree Law n. 119/2017); (**b**) changes in perception of mandatory vaccination policies after the SARS-CoV-2 pandemic.

Overall, 84.5% (*n* = 4217) of the participants agreed with the Law n.119/2017 [17], with strong agreement reported by 42.9% of them; 9.4% (*n* = 469) were undecided, while 6.1% (*n* = 307) disagreed—strongly in 2.2% (*n* = 108) of the cases (Figure 1a). When asked the same question after experiencing the COVID-19 pandemic, most of the parents/guardians disagreed (*n* = 2545, 51%) or strongly disagreed (*n* = 1698, 34%) (Figure 1b).

## 4. Discussion

We report the results of a survey performed in a large cohort of parents/guardians assessing the rate of vaccine hesitancy (VH) toward potential COVID-19 vaccination in their children aged 0–18 years old, and its determinants.

Our study, including 5054 parents/guardians of the Metropolitan city of Bologna in the north of Italy, observed a 40% rate of VH, significantly higher if compared to the general Italian population (22.7–29.1%) or university students (13.9%) [19,20,21,22].

Worldwide mass vaccination is envisaged as key strategy to mitigate the effects of the COVID-19 pandemic [23]. Therefore, vaccines should be safe and effective for all age groups, including children [24]. Moreover, the vaccination of children would produce direct and, even more, indirect effects on the reduction of social and economic burdens due to the disease, since children are often asymptomatic carriers (i.e., reduce potential severe disease complication in children and the transmission to other groups of the population; re-opening of educational institutions, work places, and leisure activities) [25,26,27].

Three types of COVID-19 vaccines (i.e., Pfizer-BioNTech, Moderna, and Oxford-AstraZeneca) are currently being tested in children [15]. Meanwhile, efforts should be made at investigating parent/guardian attitudes toward child vaccination and the reasons underlying vaccine hesitancy (VH), so as to design the most appropriate counteracting strategies [23]. Indeed, VH, described in 2015 by SAGE [16], has significantly increased in the last decade, and is currently considered a dangerous element, able to tackle the efficacy of vaccination campaigns [28].

Only a few studies have assessed parent/guardian VH toward COVID-19 vaccination in the pediatric population, showing extremely heterogeneous results (i.e., 72.6% reported by a Chinese study [29] vs. 75.8% reported by Australian investigators [30], and 48.2% in a study from UK, flanked by 40.9% of the parents/guardians responding to be “unsure but leaning towards yes” [31]). The wide heterogeneity in the rate of VH reported by the various studies is not surprising, as the phenomenon of VH is known to be of great complexity, as it results from the confluence of several demographic, socio-economic, cultural, and geographic factors [16].

A possible explanation for the higher overall rate of VH demonstrated by our study could be the perception of a low risk of severe COVID-19 disease in children, especially by younger parents/guardians, which confirmed results of previous studies regarding the higher confidence in and propensity toward vaccination of their children of parents/guardians aged ≥50 [32,33]. In addition, children’s age also influenced parents/guardians’ attitudes toward vaccination, with the rate of VH being significantly higher in children aged <14.

In line with other studies, females were more hesitant than males [20,33,34]. This could be due to the fact that men, reported to be at higher risk of severe COVID-19 complications, have a stronger risk perception for themselves and for their children, and so could be more prone to vaccination. On the other hand, women tend to experience more adverse events after vaccination, so could be more worried about potential adverse events of the vaccine in their children, and therefore are more reluctant to vaccinating their offspring [35,36]. On the other hand, differently from other studies, higher educational levels were associated with higher confidence toward vaccination in our experience [37,38,39].

Particularly interesting are the media-related data from our study: The source of information significantly influenced attitudes toward COVID-19 vaccine, and varied with educational levels. In particular, 84.4% of parents/guardians mainly relied on medical advice; personal beliefs, web/social media, and television; the opinion of religious authorities/politicians/celebrities were not taken into account by most, although people with lower levels of education were less likely to consult physicians in favor of the Web and social media. These factors may suggest that a higher level of education provides a protective effect against VH by giving more tools for decision-making, without falling prey to conspiracy theories. Moreover, as reported by other groups, those who mainly relied on Web sources and/or social media tended to be more hesitant [40,41,42,43]. Falsehoods are more widely shared by the web than by other media, with highly active, interconnected clusters of vaccine opponents around the world that strongly try to entangle with undecided clusters and dominate the Web community [44]. On the contrary, relying on TV information appeared to be a protective factor for VH. Interestingly, Ruiz et al. identified the most and least protective channels associated with VH [45].

Despite a heavy impact on school activities of policies against COVID-19, it seems that VH and disagreement with the so-called “Lorenzin” Law have increased. It would be important to investigate this increase with further studies and specifically to evaluate the effects of school policies on VH.

## 5. Conclusions

The main strengths of the study are the large cohort of enrolled subjects, the homogeneity of the geographic origin, the absence of operator influence on questionnaire-filling, and the homogeneous distribution of both children and parents/guardians for age, gender, and level of education [46]. On the other hand, some limitations have to be disclosed. First, the non-randomly selected participants were enrolled online during a screening program performed in parents/guardians of children aged 0–18, who voluntarily adhered to the study; thus, (a) it is not possible to calculate a survey participation rate, (b) it is not possible to state the confidence limits of the estimated prevalence of VH, and (c) it is not clear which subset of the parent population the survey participants represent. Additionally, the survey was carried out at the very beginning of the Italian anti-COVID-19 vaccination campaign, when the public debate on this topic was not yet politicized or polarized, so data could be not representative of the actual perception of the people.

In conclusion, parents’ or guardians’ VH toward the COVID-19 vaccine in their children was observed to be higher than for other vaccines; female gender, younger parental/children’s age, lower educational level, and referral to the Web/social media were found to significantly increase the risk of VH. Although preliminary, these data could help in designing target strategies to implement adherence to vaccination campaigns, with special regard to Web-based information.

## Figures and Tables

**Table 1:** Main features of the sample:

(**a**)									
**Age** (**Years**)	**Primary School Degree**		**High School Degree**		**University Degree**		**Post-University Master Degree**		**Total**
	**(Grades 1–8)**		**(Grades 9–13)**						**N** (**%**)
	**M**	**F**	**M**	**F**	**M**	**F**	**M**	**F**	
≤29	3	21	4	41	3	15	2	0	89 (1.8)
30–39	22	82	48	369	53	264	22	82	942 (18.9)
40–49	80	175	252	973	171	765	62	286	2764 (55.4)
≥50	50	60	216	372	147	236	34	83	1198 (24.0)
Total N (%)	155 (3.1)	338 (6.8)	520 (10.4)	1755 (35.1)	374 (7.5)	1280 (25.6)	120 (2.4)	451 (9.0)	4993
				(**b**)					
				**Age**	**N** (**%**)				
				0–5	633 (12.7)				
				06-ott	1807 (36.2)				
				nov-13	1312 (26.3)				
				≥14	1241 (24.9)				
				Total	4993				

(**a**) Parents/guardians; (**b**) children.

**Table 2:** Source of information and propensity toward COVID-19 vaccination.

Information Source	Propensity toward Vaccination	
	Yes	No
Medical advice	4214 (84.4)	779 (15.6)
Personal beliefs	1432 (28.7)	3557 (71.2)
Web/Social Media	362 (7.3)	4631 (92.7)
Television	295 (5.9)	4698 (94.1)
Opinion of politics/religious authorities/celebrities	64 (1.3)	4929 (98.7)

**Table 3:** Predictors of vaccine hesitancy (VH) according to multivariate analysis ^1^.

Vaccine Hesitancy		OR	SE	*p*-Value	95% C.I.
Parental Age	≤29	1.89	0.51	0.019	1.11–3.23
	30–39	1.51	0.18	0.001	1.19–1.93
	40–49	1.34	0.12	0.001	1.12–1.61
	≥50	1			
Parental Gender		1.62	0.13	<0.001	1.37–1.91
Parental Educational Level	Primary school qualification	1.47	0.22	0.011	1.09–1.96
	High school qualification	1.31	0.15	0.020	1.04–1.64
	University degree	1.24	0.15	0.071	0.98–1.56
	Post-university Master’s degree	1			
Children Age	0–5	1.57	0.21	0.001	1.21–2.04
	6–10	1.71	0.17	<0.001	1.40–2.07
	11–13	1.53	0.15	<0.001	1.26–1.85
	≥14	1			
Determinants (SAGE)	Medical advice	0.63	0.07	<0.001	0.51–0.78
	Personal beliefs	0.74	0.07	0.001	0.62–0.88
	Web/Social Media	1.86	0.27	<0.001	1.41–2.47
	Television	0.71	0.11	0.031	0.52–0.97
Dislike of Mandatory Vaccination Policies		2.62	0.12	<0.001	2.41–2.86
Change in Policies Perception		1.5	0.05	<0.001	1.40–1.61

^1^ All the listed variables were included in the model. Adherence to previous vaccinations variable was excluded by the backward stepwise logistic analysis.

**Table 4:** Sources of information chosen according to the level of parental/guardian education.

		Primary School Degree	High School Degree	University Degree	Post-University Master Degree	Total
Medical Advice	No	135 (27.4)	375 (16.5)	211 (12.8)	57 (10.0)	778 (15.6)
	Yes	358 (72.6)	1900 (83.5)	1443 (87.2)	514 (90.0)	4215 (84.4)
Web/Social Media	No	448 (90.9)	2107 (92.7)	1538 (93.0)	537 (94.0)	4631 (92.7)
	Yes	45 (9.1)	167 (7.3)	116 (7.0)	34 (6.0)	362 (7.3)

## Supplementary Materials

The following are available online at https://www.mdpi.com/article/10.3390/vaccines9040366/s1, File S1: Survey on VH and Perception of Vaccination Policies.
