# Supplementary material for: Would Parents Get Their Children Vaccinated Against SARS-CoV-2? Rate and Predictors of Vaccine Hesitancy According to a Survey over 5000 Families from Bologna, Italy
Source: Vaccines (Basel). 2021 Apr 10;9(4):366. doi: 10.3390/vaccines9040366 (PMC8069076; doi:10.3390/vaccines9040366)
Supplement: Supplementary file 1 [file vaccines-09-00366-s001.pdf]

Survey on VH and Perception of Vaccination Policies

Personal information

- Parents/Guardians' Age (years
- Parents/Guardians' Gender
- Parents/Guardians' Educational level
- Children's age
- Children's school grade

Personal experiences

1) **The Italian government ("Lorenzin" Law) in 2017 in order to prevent communicable diseases and reduce public health concerns increased the number of mandatory vaccines from four to ten in order to enrol in nurseries and kindergartens. What did you think?**
    - I totally agree
    - I agree
    - Neutral
    - I disagree
    - I totally disagree

2) **My perception of the policy of mandatory vaccinations has changed in the light of the current SARS-CoV-2 (COVID) pandemic?**
    - I totally agree
    - I agree
    - Neutral
    - I disagree
    - I totally disagree

3) **Will you vaccinate your child for SARS-CoV-2 (COVID)? (*Please note that currently a paediatric vaccination for SARS-CoV-2 is not available)**
    - Yes
    - I don't know
    - No

4) **Did you vaccinate your child for all the vaccinations that were required?**
    - Yes
    - No
    - Not all, just a few

5) **How did you decide whether or not to vaccinate your child?** [select all that apply]
    - Checking the Internet/Social Media
    - Listening to the TV
    - Following people (politicians, religious, influencers ...)
    - With advice from doctors/healthcare staff
    - Personal beliefs
